# Eliminating the isoleucine biosynthetic pathway to reduce competitive carbon outflow during isobutanol production by *Saccharomyces cerevisiae*

**DOI:** 10.1186/s12934-015-0240-6

**Published:** 2015-04-29

**Authors:** Kengo Ida, Jun Ishii, Fumio Matsuda, Takashi Kondo, Akihiko Kondo

**Affiliations:** Department of Chemical Science and Engineering, Graduate School of Engineering, Kobe University, 1-1 Rokkodai, Nada, Kobe, 657-8501 Japan; Organization of Advanced Science and Technology, Kobe University, 1-1 Rokkodai, Nada, Kobe, 657-8501 Japan; Department of Bioinformatic Engineering, Graduate School of Information Science and Technology, Osaka University, Suita, Osaka 565-0871 Japan; RIKEN Biomass Engineering Program, 1-7-22 Suehiro, Tsurumi, Yokohama, 230-0045 Japan; Present address: Faculty of Engineering, Hokkaido University, N13W8, Sapporo, 060-8628 Japan

**Keywords:** Isobutanol, Isoleucine, Gene deletion, Competitive pathway, *ILV1*, *Saccharomyces cerevisiae*

## Abstract

**Background:**

Isobutanol is an important biorefinery target alcohol that can be used as a fuel, fuel additive, or commodity chemical. Baker’s yeast, *Saccharomyces cerevisiae*, is a promising organism for the industrial manufacture of isobutanol because of its tolerance for low pH and resistance to autolysis. It has been reported that gene deletion of the pyruvate dehydrogenase complex, which is directly involved in pyruvate metabolism, improved isobutanol production by *S. cerevisiae*. However, the engineering strategies available for *S. cerevisiae* are immature compared to those available for bacterial hosts such as *Escherichia coli*, and several pathways in addition to pyruvate metabolism compete with isobutanol production.

**Results:**

The isobutyrate, pantothenate or isoleucine biosynthetic pathways were deleted to reduce the outflow of carbon competing with isobutanol biosynthesis in *S. cerevisiae*. The judicious elimination of these competing pathways increased isobutanol production. *ILV1* encodes threonine ammonia-lyase, the enzyme that converts threonine to 2-ketobutanoate, a precursor for isoleucine biosynthesis. *S. cerevisiae* mutants in which *ILV1* had been deleted displayed 3.5-fold increased isobutanol productivity. The Δ*ILV1* strategy was further combined with two previously established engineering strategies (activation of two steps of the Ehrlich pathway and the transhydrogenase-like shunt), providing 11-fold higher isobutanol productivity as compared to the parent strain. The titer and yield of this engineered strain was 224 ± 5 mg/L and 12.04 ± 0.23 mg/g glucose, respectively.

**Conclusions:**

The deletion of competitive pathways to reduce the outflow of carbon, including *ILV1* deletion, is an important strategy for increasing isobutanol production by *S. cerevisiae*.

## Background

The rise in oil prices and environmental concerns has heightened interest in the microbial production of fuels and chemicals from sugar feedstocks produced from renewable biomass. Branched higher alcohols are both representative promising next-generation biofuels and building blocks for producing a variety of chemicals [1,2]. In particular, isobutanol can be used as a fuel, fuel additive, and a commodity chemical, and thus is an important biorefinery target alcohol. Furthermore, isobutanol has attractive properties, including lower toxicity and higher octane value than its straight-chain counterpart [3].

Metabolically engineered microbial strains for producing isobutanol have been developed by introducing parts of the Ehrlich pathway into bacterial hosts such as *Escherichia coli*, *Corynebacterium glutamicum*, *Clostridium cellulolyticum*, and *Bacillus subtilis* [3-8]. In these recombinant strains, an intermediate of valine biosynthesis, 2-ketoisovalerate, is converted into isobutanol through isobutyraldehyde by two steps of the Ehrlich pathway involving 2-keto acid decarboxylase (2-KDC) and alcohol dehydrogenase (ADH) [4]. In bacterial hosts, metabolic pathway engineering, including overexpression of several enzymes, has resulted in increased isobutanol production levels [4-8]. In *E. coli* in particular, additional metabolic modifications, such as deletion of competing pathways and resolving cofactor imbalance, have provided quite high yields of isobutanol (21.2 g/L and 13.4 g/L; 76% and 100% of theoretical maximum yields, respectively) [9,10].

Baker’s yeast, *Saccharomyces cerevisiae*, is a microorganism traditionally used in the brewing industry [11]. It is also a promising host organism for the industrial manufacture of biofuels and chemicals because of its significant potential for the bulk-scale production of various fermentation compounds. Furthermore, *S. cerevisiae* is tolerant of low pH (used to reduce the risk of contamination), and robust towards autolysis (allowing long-term, repeated or continuous fermentation) [12-14].

Yeasts naturally produce isobutanol and have been studied for a long time [15-17]. Isobutanol-high-producing yeasts were initially developed using strategies similar to those used for bacteria. For example, *kivd* from *Lactococcus lactis* (2-KDC) and *ADH6* from *S. cerevisiae* (ADH) were expressed to construct parts of the Ehrlich pathway in the cytosol of baker’s yeast cells [13,14]. Isobutanol production was further increased by either activating the innate valine biosynthetic pathway in the mitochondria [13,14] or by constructing an artificial pathway in the cytosol by expressing the N-terminal truncated forms of acetolactate synthase (ALS; encoded by *ILV2*), ketol-acid reductoisomerase (KARI; encoded by *ILV5*), and dihydroxyacid dehydratase (DHAD; encoded by *ILV3*) [18,19]. Recently proposed strategies are to artificially co-localize 2-KDC and ADH in the mitochondria to compartmentalize parts of the Ehrlich pathway [20], and to artificially activate the transhydrogenase-like shunt comprising pyruvate carboxylase, malate dehydrogenase and malic enzyme to compensate for cofactor imbalances [21].

The elimination or attenuation of competing pathways is another effective strategy for improving isobutanol production by *S. cerevisiae*. For example, deletion of the major isozyme of pyruvate decarboxylase (encoded by *PDC1*), which catalyzes the conversion of pyruvate to acetaldehyde, results in increased isobutanol production [14]. More recently, deletion of either *PDA1, PDB1, LAT1* or *LPD1* (which together encode the pyruvate dehydrogenase complex, responsible for converting pyruvate to acetyl-CoA), led to much higher isobutanol production [21]. This was verified by screening the catalytic enzymes directly involved in pyruvate metabolism [21]. However, strategies for engineering *S. cerevisiae* remain poorly developed compared to those for bacterial hosts such as *E. coli* [22]. Consequently, there may be several pathways, other than pyruvate conversion pathways, that compete with isobutanol production in *S. cerevisiae*.

**Figure 1:**
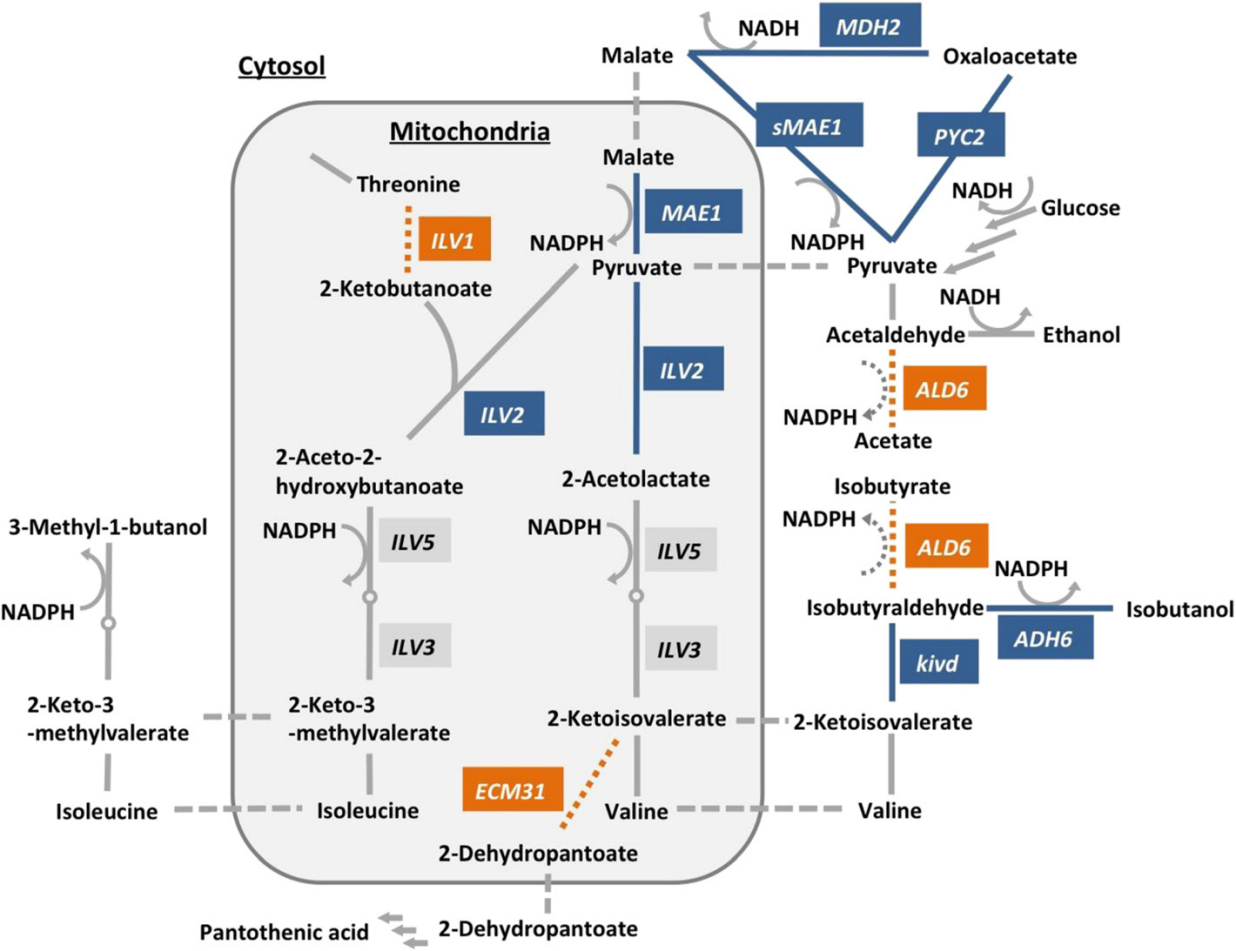
Metabolic map of isobutanol biosynthesis by *S. cerevisiae*. The genes deleted to prevent competitive pathways are indicated by white letters on orange backgrounds (*ALD6*, *ECM31* and *ILV1*). The overexpressed genes are indicated by white letters on blue backgrounds (*kivd*, *ADH6*, *ILV2*, *PYC2*, *MDH2*, *MAE1* and *sMAE1*).

In this study, we deleted the isobutyrate, pantothenate, and isoleucine biosynthetic pathways in *S. cerevisiae* to reduce carbon outflow competing with isobutanol biosynthesis (Figure 1). The judicious elimination of these competing pathways should result in increased isobutanol production. In addition, it should be possible to combine the elimination of competing pathways with previous strategies for enhancing the isobutanol biosynthetic pathway and compensating for cofactor imbalances, thereby further increasing isobutanol production.

## Results and discussion

### Strategy to reduce the competitive outflow of carbon during isobutanol biosynthesis

Several enzymes have broad substrate specificities; for example, aldehyde dehydrogenase can catalyze the oxidation of several kinds of aldehydes such as acetaldehyde, isobutyraldehyde, isopentaldehyde, and 2-methyl-butyraldehyde [23,24]. Cytosolic aldehyde dehydrogenase is encoded by *ALD6* and normally converts acetaldehyde to acetate, but can also convert other aldehydes to carboxylates such as isobutyraldehyde to isobutyrate [24]. Thus, the deletion of *ALD6* could increase the amount of isobutyraldehyde available for isobutanol biosynthesis (Figure 1).

A primary intermediate in isobutanol biosynthesis, 2-ketoisovalerate, also functions as an initial substrate in pantothenic acid biosynthesis [25]. 3-Methyl-2-oxobutanoate hydroxymethyltransferase, encoded by *ECM31*, catalyzes the first step in pantothenic acid biosynthesis. Consequently, deletion of *ECM31* could prevent the diversion of 2-ketoisovalerate into the pantothenate pathway (Figure 1).

Isoleucine and valine biosynthesis are parallel pathways catalyzed by the same enzymes, ALS, KARI and DHAD (encoded by *ILV2*, *ILV5* and *ILV3*) [26]. The intermediate of isoleucine biosynthesis, 2-aceto-2-hydroxybutanoate, is synthesized from pyruvate and 2-ketobutanoate by ALS catalysis. It is expected that the prevention of isoleucine biosynthesis would stop the competitive outflow of carbon from the pyruvate pathway to the isoleucine pathway, and additionally should consolidate the activities of three enzymes (ALS, KARI and DHAD) into valine and isobutanol biosynthesis. *ILV1* encodes threonine ammonia-lyase, the enzyme that converts threonine to 2-ketobutanoate, a precursor for isoleucine biosynthesis. Thus, the deletion of *ILV1* should specifically prevent carbon flux into the isoleucine pathway (Figure 1).

### Isobutanol production by single-gene knockout strains

**Figure 2:**
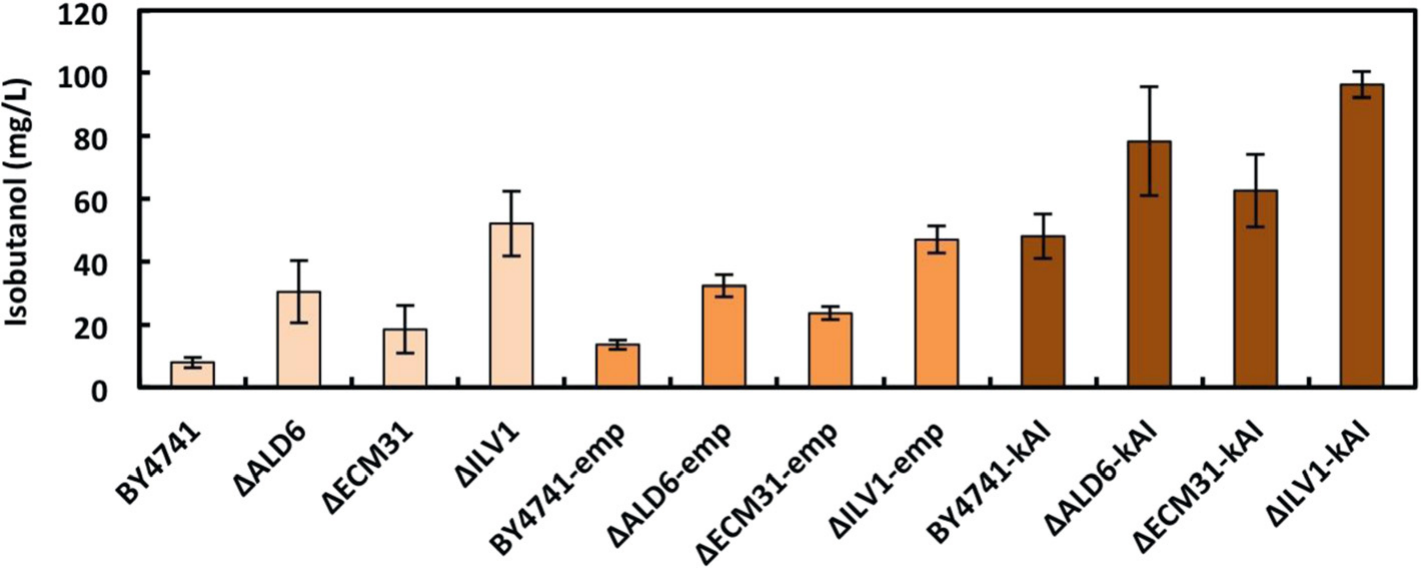
Isobutanol production by BY4741 single-gene knockout strains. BY4741*-emp* and Δ*XXXX-emp* are the control strains harboring the pATP426 empty vector. BY4741*-kAI* and Δ*XXXX-kAI* harbor the pATP426-kivd-ADH6-ILV2 plasmid for enhancing isobutanol biosynthesis. Cells were inoculated at an OD_600_ of 2 and grown in SD minimal or selectable media. For Δ*ILV1* strains, 60 mg/L of isoleucine was added to the SD medium. The concentration of isobutanol in the medium of each culture after 2 days of fermentation was determined using GC-MS. Each data point represents the mean (SD) values obtained from 3 replicate fermentations.

The effects of eliminating the isobutyrate, pantothenate, and isoleucine biosynthetic pathways were determined using the BY4741 parent strain [27] and single-gene knockout mutants (BY4741Δ*ALD6*, BY4741Δ*ECM31* and BY4741Δ*ILV1*) [28] (Table 1). All strains were inoculated at an optical density at 600 nm (OD_600_) of 2 and grown in synthetic dextrose (SD) minimal or selectable media under semi-anaerobic conditions. For BY4741Δ*ILV1* strain, 60 mg/L of isoleucine was added to the SD medium. Isobutanol concentrations in the media after 2 days of fermentation were determined by gas chromatography mass spectrometry (GC-MS). As shown in Figure 2, all gene knockout strains showed increased isobutanol production compared to the parent BY4741 strain: the *ALD6*, *ECM31* and *ILV1* knockout strains respectively showed 2.4-, 1.7- and 3.5-fold higher productivities of isobutanol than the parent strain.

**Table 1:** **Yeast strains used in this study**

**Strains**	**Genotypes**
BY4741	*MAT* **a** *his3*Δ*1 leu2*Δ*0 met15*Δ*0 ura3*Δ*0*
BY4741Δ*ALD6*	BY4741 *ald6*Δ
BY4741Δ*ECM31*	BY4741 *ecm31*Δ
BY4741Δ*ILV1*	BY4741 *ilv1*Δ
BY4741*-emp*	BY4741/pATP426
BY4741Δ*ALD6-emp*	BY4741Δ*ALD6*/pATP426
BY4741Δ*ECM31-emp*	BY4741Δ*ECM31*/pATP426
BY4741Δ*ILV1-emp*	BY4741Δ*ILV1*/pATP426
BY4741-*kAI*	BY4741/pATP426-kivd-ADH6-ILV2
BY4741Δ*ALD6*-*kAI*	BY4741Δ*ALD6*/pATP426-kivd-ADH6-ILV2
BY4741Δ*ECM31*-*kAI*	BY4741Δ*ECM31*/pATP426-kivd-ADH6-ILV2
BY4741Δ*ILV1*-*kAI*	BY4741Δ*ILV1*/pATP426-kivd-ADH6-ILV2
YPH499	*MAT* **a** *ura3-52 lys2-801 ade2-101 trp1-*Δ*63 his3-*Δ*200 leu2-*Δ*1*
YPH499Δ*ILV1*	YPH499 *ilv1*Δ
YPH499Δ*ILV1-emp*	YPH499Δ*ILV1*/pATP426
YPH499Δ*ILV1*-*kAI*	YPH499Δ*ILV1*/pATP426-kivd-ADH6-ILV2/pATP423
YPH499Δ*ILV1*-*kAI*-*MAE1*	YPH499Δ*ILV1*/pATP426-kivd-ADH6-ILV2/pATP423-MAE1
YPH499Δ*ILV1*-*kAI*-*PMsM*	YPH499Δ*ILV1*/pATP426-kivd-ADH6-ILV2/pATP423-PMsM

Next, the pATP426-kivd-ADH6-ILV2 plasmid, which carries three genes (*kivd*, *ADH6* and *ILV2*) [21], was introduced into the parent and each knockout strain to enhance isobutanol biosynthesis. The generated strains harboring pATP426-**k**ivd-**A**DH6-**I**LV2 were designated as BY4741-*kAI*, BY4741Δ*ALD6*-*kAI*, BY4741Δ*ECM31*-*kAI* and BY4741Δ*ILV1*-*kAI* (Tables 1 and 2). To generate comparative mock strains as controls, parent BY4741, BY4741Δ*ALD6*, BY4741Δ*ECM31* and BY4741Δ*ILV1* were transformed with an **emp**ty vector (pATP426) [29] to provide BY4741*-emp*, BY4741Δ*ALD6-emp*, BY4741Δ*ECM31-emp* and BY4741Δ*ILV1*-*emp*, respectively (Tables 1 and 2). All transformants were grown similarly in SD selectable medium. Isobutanol production by the control strain (BY4741*-emp*) was similar to that of the knockout strains lacking either plasmid (Figure 2). In contrast, the strains engineered for enhanced isobutanol biosynthesis (BY4741Δ*XXXX*-*kAI*) showed 2–3-fold higher isobutanol productivity than the corresponding control strain (Figure 2). The pattern in increase of isobutanol production on each gene deletion was similar to that observed using empty plasmids. The most effective gene deletion was Δ*ILV1*, and the BY4741Δ*ILV1*-*kAI* strain produced 96 ± 4 mg/L isobutanol. This concentration of isobutanol produced by BY4741Δ*ILV1*-*kAI* was 6.9-fold higher than that obtained with the BY4741*-emp* control strain. Thus, we focused on *ILV1* deletion in the following experiments.

**Table 2:** **Plasmids used in this study**

**Plasmid**	**Description**	**Source or reference**
pATP426	Yeast three gene expression vector containing *ADH1*, *TDH3*, and *PGK1* promoters, 2 *μ* origin, *URA3* marker, no expression (control plasmid)	Ishii et al., 2014 [29]
pATP426-kivd-ADH6-ILV2	pATP426, co-expression of *L. lactis kivd*, *S. cerevisiae ADH6,* and *ILV2* genes	Matsuda et al., 2013 [21]
pATP423	Yeast three gene expression vector containing *ADH1*, *TDH3*, and *PGK1* promoters, 2 *μ* origin, *HIS3* marker, no expression (control plasmid)	Ishii et al., 2014 [29]
pATP423-MAE1	pATP423, expression of *S. cerevisiae MAE1* gene	Matsuda et al., 2013 [21]
pATP423-PMsM	pATP423, co-expression of *S. cerevisiae sMAE1*, *MDH2*, and *PYC2* genes	Matsuda et al., 2013 [21]

### Optimization of isoleucine supplementation for isobutanol production in *ILV1*-deleted YPH499 strain

We previously demonstrated that YPH499 strain [30] displayed higher isobutanol productivity than BY4741 strain [21]; consequently we constructed *ILV1*-deleted YPH499 (YPH499Δ*ILV1*) using the *URA3* marker recycling method [31] (Table 1). The strain produced a slightly higher amount of isobutanol than BY4741Δ*ILV1* in SD minimal medium (data not shown). Therefore, YPH499Δ*ILV1* was used in subsequent experiments.

**Figure 3:**
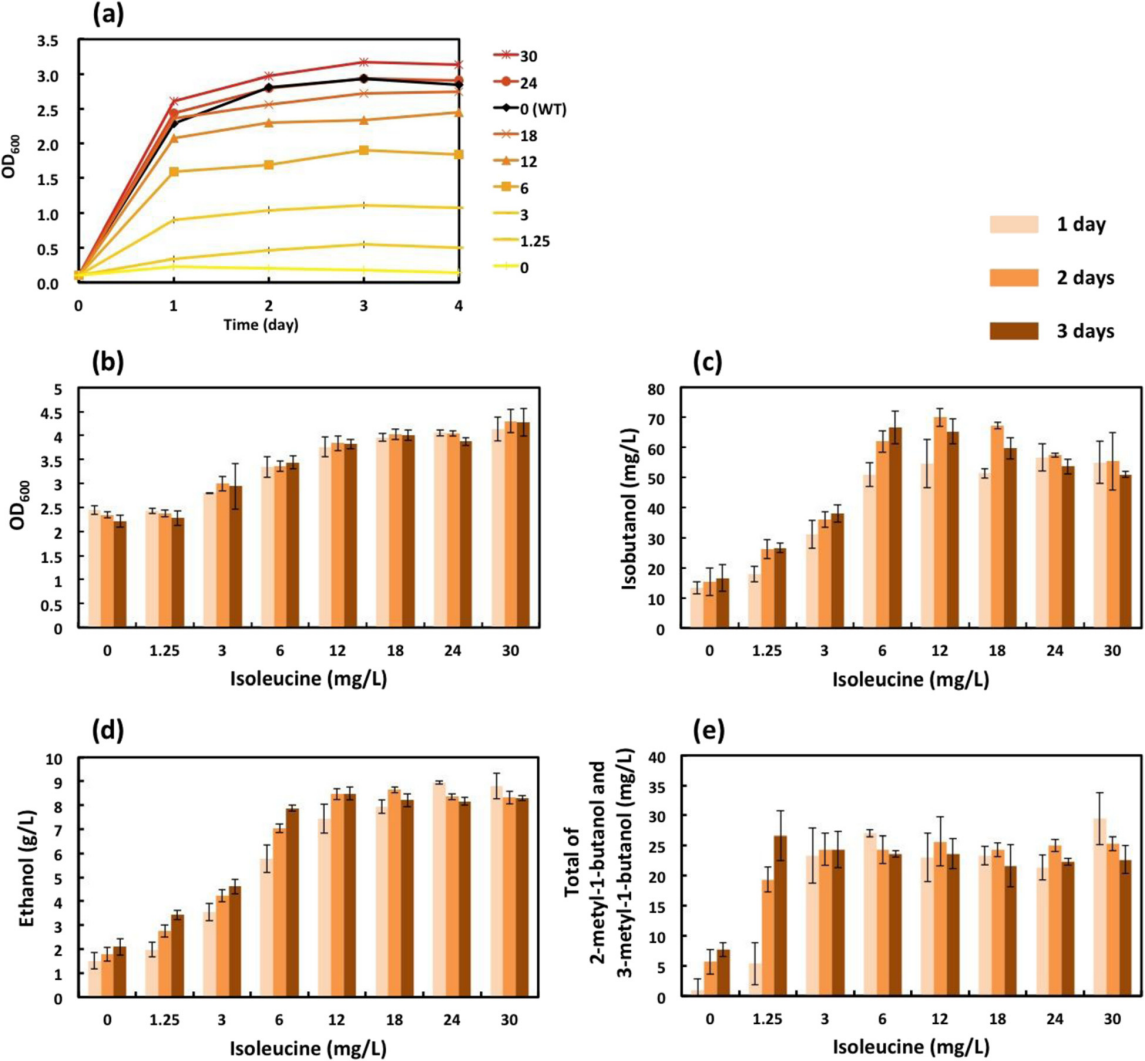
Time course of cultivation of and fermentation by YPH499Δ*ILV1* strain in isoleucine-containing media. **(a)** YPH499Δ*ILV1* was inoculated at an OD_600_ of 0.1 and cultured in SD minimal medium containing 0, 1.25, 3, 6, 12, 18, 24 or 30 mg/L isoleucine. Cell growth was determined by measuring OD_600_ using a spectrophotometer. **(b)(c)(d)(e)** YPH499Δ*ILV1* was inoculated at an OD_600_ of 2 and grown in SD minimal medium containing different concentrations of isoleucine. The cell growth was determined by measuring the OD_600_ using a spectrophotometer, and the concentrations of isobutanol, ethanol, and the total of 2-methyl-1-butanol and 3-methyl-1-butanol, in the media were determined using GC-MS. Each data point represents the mean (SD) values obtained from 3 replicate fermentations.

The *ILV1-*deleted strain was an isoleucine auxotroph, since the *ILV1* deletion stops 2-ketobutanoate biosynthesis, rendering the yeast incapable of isoleucine biosynthesis (Figure 1) [26]. YPH499Δ*ILV1* was therefore cultured in SD minimal medium containing different concentrations of isoleucine (0, 1.25, 3, 6, 12, 18, 24, 30 mg/L) to determine the optimal concentration for isobutanol production. YPH499Δ*ILV1* yeast cells were inoculated at an OD_600_ of 0.1 into SD minimal medium supplemented with each concentration of isoleucine, and the growth was monitored daily for 4 days (Figure 3a). No cell growth was observed in the isoleucine-free medium, whereas cell growth improved with increasing isoleucine concentration. Cell growth comparable to the parent YPH499 strain (without isoleucine supplementation) was observed using medium containing 24 mg/L isoleucine.

Next, YPH499Δ*ILV1* was inoculated at an OD_600_ of 2 in SD minimal media supplemented with the different concentrations of isoleucine; cell growth, and the concentration of product (isobutanol) and by-products (ethanol, 2-methyl-1-butanol and 3-methyl-1-butanol) in the medium, were determined after 1–3 days of fermentation using a spectrophotometer and GC-MS, respectively. 2-Methyl-1-butanol and 3-methyl-1-butanol could not be separated by our GC-MS method [14]. Their concentration at each time point was calculated as the total concentration of both compounds, although addition of isoleucine might be more likely to increase 2-methyl-1-butanol production [32]. The growth of YPH499Δ*ILV1* plateaued in the presence of 12–30 mg/L isoleucine (Figure 3b). The concentrations of isobutanol and ethanol plateaued in medium containing 12 mg/L isoleucine (Figure 3c, d), whereas the total concentration of 2-methyl-1-butanol and 3-methyl-1-butanol plateaued at 3 mg/L isoleucine (Figure 3e). The highest concentration of isobutanol obtained was 70 ± 3 mg/L after 2 days fermentation in the presence of 12 mg/L isoleucine.

In terms of costs for commercial application, it should rein in the amount of additive isoleucine. For this purpose, it might be required to supply isoleucine from pretreated biomass or to tune the Ilv1 expression level in the future.

### Improvement of isobutanol production by YPH499Δ*ILV1* strain

Isobutanol biosynthesis requires NADPH as a cofactor for the reaction catalyzed by KARI (Ilv5) and ADH (Adh6); consequently, regeneration of NADPH is an important factor for increasing the amount of isobutanol. Thus, the regeneration of NADPH is an important factor for improving isobutanol production [10,21]. A transhydrogenase-like shunt composed of pyruvate carboxylase (PYC), malate dehydrogenase (MDH), and malic enzyme (MAE) has been developed to regenerate NADPH in yeast [33,34] and used to resolve the redox imbalance in xylose fermentation [35]. Through this shunt, pyruvate is sequentially converted to oxaloacetate, malate and pyruvate by Pyc2, Mdh2 and Mae1 in *S. cerevisiae* (Figure 1). Because the cofactor preferences of Mdh2 and Mae1 are NADH and NADP^+^, respectively, one NADH is consumed and one NADPH is regenerated during each cycle of this shunt pathway [33-35]. This transhydrogenase-like shunt has also been used to improve isobutanol production [21]. Notably, two versions of malic enzyme (Mae1) with distinct localizations were utilized for constructing two versions of the shunt pathway. One is the original yeast protein Mae1, which localizes in the mitochondria, and the other is N-terminal truncated Mae1 (sMae1), which localizes in the cytosol [36]. Because the first version, original Mae1, regenerates NADPH in the mitochondria, the cofactor imbalance in the KARI (Ilv5) reaction should be improved (Figure 1). The second version, the truncated Mae1 (sMae1), should reduce the cofactor imbalance in the ADH (Adh6) reaction in the cytosol (Figure 1). Since the yeast originally has the three enzymes Pyc2, Mdh2 and Mae1 but lacks sMae1, the introduction of a transhydrogenase-like shunt should be a viable strategy even if one of Pyc2, Mdh2 or Mae1 is overexpressed. In this study, we tested the effect of the overexpression of *MAE1* alone, and the co-overexpression of *MAE1* with *PYC2*, *MDH2* and *sMAE1*. This choice was based on the previous finding that the highest isobutanol productivity by YPH499 was obtained using the recombinant strain overexpressing *kivd*, *ADH6* and *ILV2* [21].

**Figure 4:**
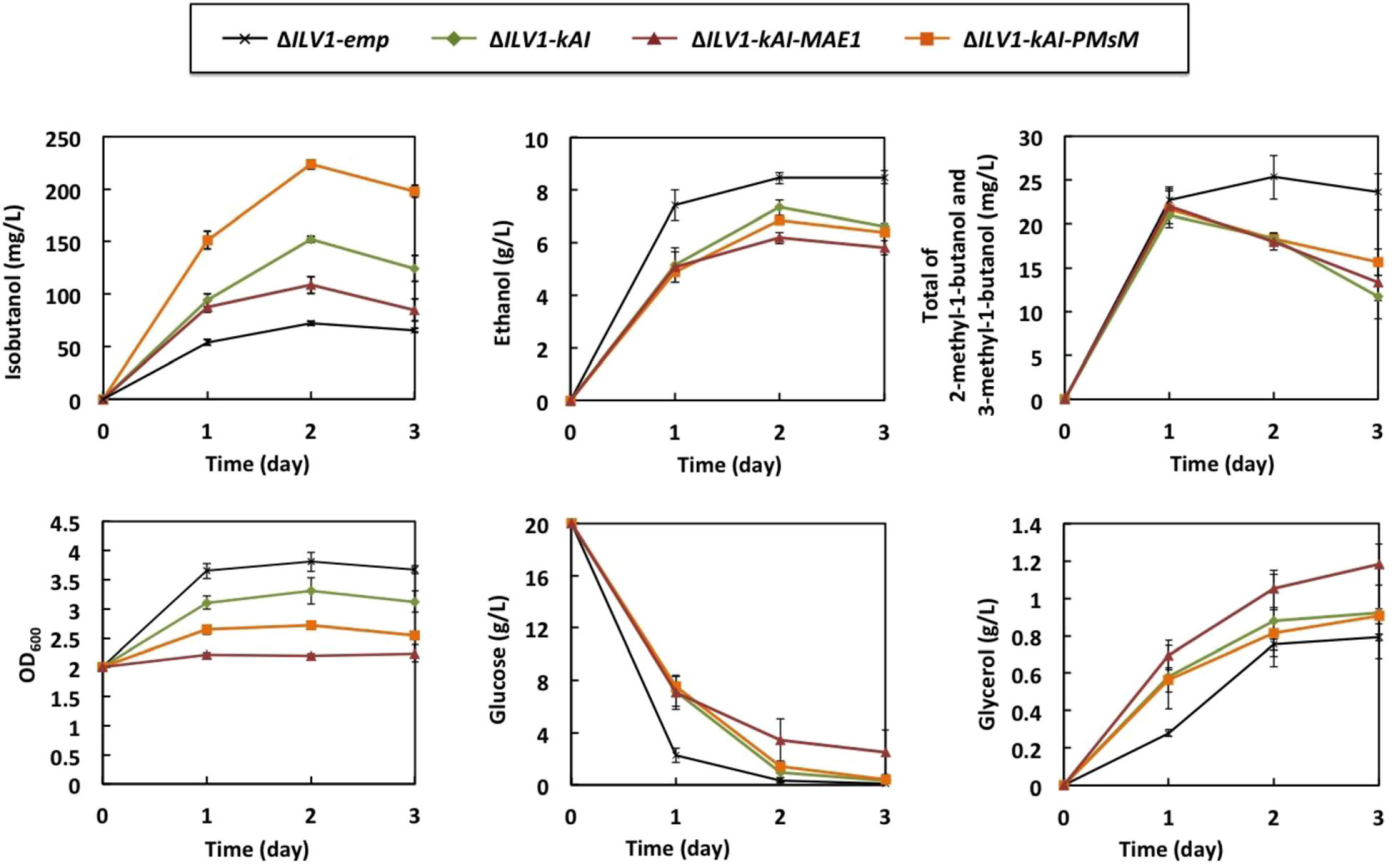
Time course of fermentation by the YPH499Δ*ILV1* transformants. Δ*ILV1-emp* indicates the strain harboring the pATP426 empty vector and Δ*ILV1-kAI* indicates the strain harboring the pATP423 empty vector and pATP426-kivd-ADH6-ILV2 plasmid for enhancing isobutanol biosynthesis. Δ*ILV1-kAI*-PMsM and Δ*ILV1-kAI-MAE1* indicate the strains harboring pATP426-kivd-ADH6-ILV2 and pATP423-PMsM, and pATP426-kivd-ADH6-ILV2 and pATP423-MAE1, for activating the cytosolic or mitochondrial transhydrogenase-like shunt, respectively. The transformants were inoculated at an OD_600_ of 2 and grown in SD selectable medium containing 12 mg/L isoleucine. The cell growth was determined by measuring OD_600_ using a spectrophotometer. The concentrations of isobutanol, ethanol, and the total of 2-methyl-1-butanol and 3-methyl-1-butanol, in the media were determined using GC-MS. The concentrations of glucose and glycerol in the media were determined using HPLC. Each data point represents the mean (SD) values obtained from 3 replicate fermentations.

To generate the yeast strains overexpressing ***M****AE1* (YPH499Δ*ILV1*-*kAI*-*MAE1*) and ***P****YC2*, ***M****DH2* and ***sM****AE1* (YPH499Δ*ILV1*-*kAI*-*PMsM*), pATP423-MAE1 and pATP423-PMsM [21] were respectively introduced into YPH499Δ*ILV1* along with pATP426-kivd-ADH6-ILV2 (Tables 1 and 2). The comparative strains YPH499Δ*ILV1-emp* harboring pATP426, YPH499Δ*ILV1*-*kAI* harboring pATP423, and pATP426-kivd-ADH6-ILV2 were also generated (Tables 1 and 2). Fermentation by these four strains was initiated at an OD_600_ of 2 in SD selectable medium containing 12 mg/L isoleucine. Figure 4 shows the time course change in several fermentation products in the medium. YPH499Δ*ILV1*-*kAI* and YPH499Δ*ILV1*-*kAI*-*PMsM* produced 153 ± 3 mg/L and 224 ± 5 mg/L of isobutanol, respectively, a 2.1- and 3.1-fold increase compared to YPH499Δ*ILV1-emp*. These increases were comparable to increases observed previously [21], suggesting that the transhydrogenase-like shunt helped maintain the NADPH supply in the cytosol. It is also worth noting that the isobutanol production level of YPH499Δ*ILV1*-*kAI*-*PMsM* was 11-fold higher than that of the parent YPH499 strain. However, YPH499Δ*ILV1*-*kAI*-*MAE1* strain, which overexpressed mitochondrial Mae1, showed lower isobutanol production compared to YPH499Δ*ILV1*-*kAI* (Figure 4), as well as lower ethanol production and no cell growth during fermentation. Since the transhydrogenase-like shunt could drastically change the balance of coenzymes and perturb metabolic flow inside the cell, the overexpressed Mae1 hampered cell growth. Otherwise, the population heterogeneity of 2 *μ* plasmids might have varied the expression levels. Consequently, the expression level and balance of Pyc2, Mdh2 and Mae1 (sMae1) or chromosomal integration are important factors for optimizing the transhydrogenase-like shunt. Various overexpression levels of these proteins were previously found to affect fermentation [21].

### Analysis of glucose and other by-products in the fermentation media

The fermentation profiles of the four constructed strains (YPH499Δ*ILV1-emp*, YPH499Δ*ILV1*-*kAI*, YPH499Δ*ILV1*-*kAI*-*MAE1* and YPH499Δ*ILV1*-*kAI*-*PMsM*) were analyzed in more detail by measuring glucose consumption and the production of other by-products (glycerol, 2-methyl-1-butanol and 3-methyl-1-butanol) using high-performance liquid chromatography (HPLC) and GC-MS (Figure 4).

The glucose consumption rates of YPH499Δ*ILV1*-*kAI*, YPH499Δ*ILV1*-*kAI*-*MAE1* and YPH499Δ*ILV1*-*kAI*-*PMsM* were lower than that of the control strain (YPH499Δ*ILV1-emp*). Consistent with this, these three strains showed similar decreases in ethanol production rates. Decreased glucose consumption and ethanol production were likely due to activation of parts of the Ehrlich pathway or introduction pATP423 vector (harboring *HIS3* marker), with concomitant improvement of isobutanol production. The isobutanol yields of YPH499Δ*ILV1-emp*, YPH499Δ*ILV1*-*kAI*, YPH499Δ*ILV1*-*kAI*-*MAE1* and YPH499Δ*ILV1*-*kAI*-*PMsM* were 3.67 ± 0.09, 8.03 ± 0.15, 6.56 ± 0.44 and 12.04 ± 0.23 mg/g glucose at 2 days, respectively. The total concentration of 2-methyl-1-butanol and 3-methyl-1-butanol produced by YPH499Δ*ILV1*-*kAI*, YPH499Δ*ILV1*-*kAI*-*MAE1* and YPH499Δ*ILV1*-*kAI*-*PMsM* decreased slightly after 1 day, while that of the control strain remained stable until the end of the fermentation. These alcohols might be reversibly converted into their corresponding aldehyde following the attenuation of glycolysis. Glycolysis would be suppressed due to glucose depletion, caused by the need to supply NADPH (Figure 1). The growth of all three strains was clearly lower than that of the control strain; the degree of growth decrease might reflect the specific decrease in glucose consumption rate and increase in glycerol production by each strain.

## Conclusions

We investigated whether the deletion of the isobutyrate, pantothenate, or isoleucine biosynthetic pathways (deletion of *ALD6*, *ECM31* or *ILV1*, respectively) improved isobutanol production by *S. cerevisiae*. Although the deletions of *ILV1* and *ALD6* have been mentioned in the patents (US8828694 and US20110201073), this is the first research paper that the effects of these gene deletions were examined closely. The deletion of each pathway increased isobutanol production, with the *ILV1* knockout being the most effective. The *ILV1* knockout prevented the competitive outflow of carbon from glucose into isoleucine biosynthesis; consequently, isobutanol biosynthesis was enhanced in isoleucine-supplemented medium. Thus, the deletion of competitive pathways for reducing carbon outflow into unproductive pathways is an important strategy for the production of target chemicals by *S. cerevisiae*.

## Methods

### Yeast strains and transformation

*S. cerevisiae* YPH499 (*MAT***a***ura3-52 lys2-801 ade2-101 trp1-*Δ*63 his3-*Δ*200 leu2-*Δ*1*) [30], BY4741 (*MAT***a***his3*Δ*1 leu2*Δ*0 met15*Δ*0 ura3*Δ*0*) [27] and BY4741 single-gene deletion mutants (knockout collections; purchased from Invitrogen) [28] were used as the host strains. Yeast transformations were carried out using the lithium acetate method [37]. The resulting strains and the utilized plasmids are listed in Tables 1 and 2. *ILV1* was deleted using the previously described *URA3* marker recycling method [31]. The primers used for *ILV1* deletion are listed in Table 3.

**Table 3:** **Primers used in this study**

**Target genes**	**Primers**
*URA3 (fw)*	5'- ttgttgttgctgctttgagttctttcttgtgtgagtgctacaagccacatttaaactaagtcaattacacaaagttagtgTTTTTTGTTCTTTTTTTTGA
*URA3 (rv)*	5'- cttagtttaaatgtggcttgGGGTAATAACTGATATAATTAAATTGAAGC
*ILV1 (fw)*	5'- AATTATATCAGTTATTACCCcaagccacatttaaactaagtcaattacacaaagttagtgaaccgacaatttactttataaatttacgcaacaacttgtt
*ILV1 (rv)*	5'- aatccttacgtctatgtttcaaaccttgttttcat

### Media, cultivation and fermentation conditions

BY4741 and the single-gene deletion mutants were cultured at 30°C in 5 mL of SD minimal medium (6.7 g/L yeast nitrogen base without amino acids and 20 g/L glucose) containing 20 mg/L histidine, 60 mg/L leucine, 20 mg/L methionine and 20 mg/L uracil. For BY4741Δ*ILV1* strain, 60 mg/L isoleucine was added. The transformants were cultured in SD selectable medium (lacking uracil for plasmid maintenance). YPH499Δ*ILV1* strain was cultured in SD minimal medium containing 40 mg/L adenine, 20 mg/L histidine, 60 mg/L leucine, 20 mg/L lysine, 40 mg/L tryptophan, 20 mg/L uracil and 0 ~ 60 mg/L isoleucine. The transformants were cultured in SD selectable medium lacking uracil and/or histidine. All yeast cells were cultured in 5 mL of medium in test tubes for 3 days. The cells were inoculated into 5 mL of fresh SD minimal or selectable medium at an OD_600_ of 0.1 to test cell growth in isoleucine-supplemented medium. For some experiments, the cells were centrifuged and washed, then inoculated at an OD_600_ of 2 to test isobutanol production. For all experiments, growth was conducted in 5 mL of medium in test tubes at 30°C, 150 opm for up to 4 days.

### Measurement of fermentation products and cell growth

The concentrations of isobutanol and ethanol, and the total concentration of 2-methyl-1-butanol and 3-methyl-1-butanol, in the fermentation media were determined using GC-MS (GCMS-QP2010 Plus; Shimadzu, Kyoto, Japan) following a previously described procedure [14]. The concentrations of glucose and glycerol were determined by HPLC (Prominence; Shimadzu), as previously described [38,39]. Cell growth was monitored by measuring OD_600_ using a spectrophotometer (UVmini-1240; Shimadzu).

## Abbreviations

ADH: Alcohol dehydrogenase
ALS: Acetolactate synthase
DHAD: Dihydroxyacid dehydratase
GC-MS: Gas chromatography mass spectrometry
HPLC: High-performance liquid chromatography
KARI: Ketol-acid reductoisomerase
2-KDC: 2-keto acid decarboxylase
MAE: Malic enzyme
MDH: Malate dehydrogenase
OD_600_: Optical density at 600 nm
PYC: Pyruvate carboxylase
SD: Synthetic dextrose
sMae1: N-terminal truncated Mae1

## References

[1] Weber C, Farwick A, Benisch F, Brat D, Dietz H, Subtil T (2010). Trends and challenges in the microbial production of lignocellulosic bioalcohol fuels. Appl Microbiol Biotechnol.

[2] Connor MR, Liao JC (2009). Microbial production of advanced transportation fuels in non-natural hosts. Curr Opin Biotechnol.

[3] Blombach B, Eikmanns BJ (2011). Current knowledge on isobutanol production with *Escherichia coli, Bacillus subtilis* and *Corynebacterium glutamicum*. Bioeng Bugs.

[4] Atsumi S, Hanai T, Liao JC (2008). Non-fermentative pathways for synthesis of branched-chain higher alcohols as biofuels. Nature.

[5] Li S, Wen J, Jia X (2011). Engineering *Bacillus subtilis* for isobutanol production by heterologous Ehrlich pathway construction and the biosynthetic 2-ketoisovalerate precursor pathway overexpression. Appl Microbiol Biotechnol.

[6] Smith KM, Cho KM, Liao JC (2010). Engineering *Corynebacterium glutamicum* for isobutanol production. Appl Microbiol Biotechnol.

[7] Yamamoto S, Suda M, Niimi S, Inui M, Yukawa H (2013). Strain optimization for efficient isobutanol production using *Corynebacterium glutamicum* under oxygen deprivation. Biotechnol Bioeng.

[8] Higashide W, Li Y, Yang Y, Liao JC (2011). Metabolic engineering of *Clostridium cellulolyticum* for production of isobutanol from cellulose. Appl Environ Microbiol.

[9] Smith KM, Liao JC (2011). An evolutionary strategy for isobutanol production strain development in *Escherichia coli*. Metab Eng.

[10] Bastian S, Liu X, Meyerowitz JT, Snow CD, Chen MM, Arnold FH (2011). Engineered ketol-acid reductoisomerase and alcohol dehydrogenase enable anaerobic 2-methylpropan-1-ol production at theoretical yield in *Escherichia coli*. Metab Eng.

[11] Donalies UE, Nguyen HT, Stahl U, Nevoigt E (2008). Improvement of *Saccharomyces* yeast strains used in brewing, wine making and baking. Adv Biochem Eng Biotechnol..

[12] Kondo A, Ishii J, Hara KY, Hasunuma T, Matsuda F (2013). Development of microbial cell factories for bio-refinery through synthetic bioengineering. J Biotechnol.

[13] Chen X, Nielsen KF, Borodina I, Kielland-Brandt MC, Karhumaa K (2011). Increased isobutanol production in *Saccharomyces cerevisiae* by overexpression of genes in valine metabolism. Biotechnol Biofuels..

[14] Kondo T, Tezuka H, Ishii J, Matsuda F, Ogino C, Kondo A (2012). Genetic engineering to enhance the Ehrlich pathway and alter carbon flux for increased isobutanol production from glucose by *Saccharomyces cerevisiae*. J Biotechnol.

[15] Guymon JF, Ingraham JL, Crowell EA (1961). The formation of n-propyl alcohol by *Saccharomyces cerevisiae*. Arch Biochem Biophys..

[16] Guymon JF, Ingraham JL, Crowell EA (1961). Influence of aeration upon the formation of higher alcohols by yeasts. Am J Enol Vitic..

[17] Dickinson JR, Harrison SJ, Hewlins MJ (1998). An investigation of the metabolism of valine to isobutyl alcohol in *Saccharomyces cerevisiae*. J Biol Chem.

[18] Matsuda F, Kondo T, Ida K, Tezuka H, Ishii J, Kondo A (2012). Construction of an artificial pathway for isobutanol biosynthesis in the cytosol of *Saccharomyces cerevisiae*. Biosci Biotechnol Biochem.

[19] Brat D, Weber C, Lorenzen W, Bode HB, Boles E (2012). Cytosolic re-localization and optimization of valine synthesis and catabolism enables increased isobutanol production with the yeast *Saccharomyces cerevisiae*. Biotechnol Biofuels.

[20] Avalos JL, Fink GR, Stephanopoulos G (2013). Compartmentalization of metabolic pathways in yeast mitochondria improves the production of branched-chain alcohols. Nat Biotechnol.

[21] Matsuda F, Ishii J, Kondo T, Ida K, Tezuka H, Kondo A (2013). Increased isobutanol production in *Saccharomyces cerevisiae* by eliminating competing pathways and resolving cofactor imbalance. Microb Cell Fact..

[22] Generoso WC, Schadeweg V, Oreb M, Boles E (2014). Metabolic engineering of *Saccharomyces cerevisiae* for production of butanol isomers. Curr Opin Biotechnol..

[23] Nagy I, Schoofs G, Compernolle F, Proost P, Vanderleyden J, De Mot R (1995). Degradation of the thiocarbamate herbicide EPTC (S-ethyl dipropylcarbamothioate) and biosafening by Rhodococcus sp. strain NI86/21 involve an inducible cytochrome P-450 system and aldehyde dehydrogenase. J Bacteriol.

[24] Saint-Prix F, Bönquist L, Dequin S (2004). *Saccharomyces cerevisiae* Functional analysis of the ALD gene family of *Saccharomyces cerevisiae* during anaerobic growth on glucose: the NADP^+^-dependent Ald6p and Ald5p isoforms play a major role in acetate formation. Microbiology.

[25] White WH, Gunyuzlu PL, Toyn JH (2001). *Saccharomyces cerevisiae* is capable of *de Novo* pantothenic acid biosynthesis involving a novel pathway of beta-alanine production from spermine. J Biol Chem.

[26] Holmberg S, Petersen JG (1988). Regulation of isoleucine-valine biosynthesis in *Saccharomyces cerevisiae*. Curr Genet.

[27] Brachmann CB, Davies A, Cost GJ, Caputo E, Li J, Hieter P (1998). Designer deletion strains derived from *Saccharomyces cerevisiae* S288C: a useful set of strains and plasmids for PCR-mediated gene disruption and other applications. Yeast.

[28] Winzeler EA (1999). Functional characterization of the *S. cerevisiae* genome by gene deletion and parallel analysis. Science.

[29] Ishii J, Kondo T, Makino H, Ogura A, Matsuda F, Kondo A (2014). Three gene expression vector sets for concurrently expressing multiple genes in *Saccharomyces cerevisiae*. FEMS Yeast Res.

[30] Sikorski RS, Hieter P (1989). A system of shuttle vectors and yeast host strains designed for efficient manipulation of DNA in *Saccharomyces cerevisiae*. Genetics.

[31] Akada R, Kitagawa T, Kaneko S, Toyonaga D, Ito S, Kakihara Y (2006). PCR-mediated seamless gene deletion and marker recycling in *Saccharomyces cerevisiae*. Yeast..

[32] Dickinson JR, Harrison SJ, Dickinson JA, Hewlins MJ (2000). An investigation of the metabolism of isoleucine to active Amyl alcohol in *Saccharomyces cerevisiae*. J Biol Chem.

[33] Boles E, de Jong-Gubbels P, Pronk JT (1998). Identification and characterization of *MAE1*, the *Saccharomyces cerevisiae* structural gene encoding mitochondrial malic enzyme. J Bacteriol.

[34] Nissen TL, Anderlund M, Nielsen J, Villadsen J, Kielland-Brandt MC (2001). Expression of a cytoplasmic transhydrogenase in *Saccharomyces cerevisiae* results in formation of 2-oxoglutarate due to depletion of the NADPH pool. Yeast.

[35] Suga H, Matsuda F, Hasunuma T, Ishii J, Kondo A (2013). Implementation of a transhydrogenase-like shunt to counter redox imbalance during xylose fermentation in *Saccharomyces cerevisiae*. Appl Microbiol Biotechnol.

[36] Moreira Dos Santos M, Raghevendran V, Kotter P, Olsson L, Nielsen J. Manipulation of malic enzyme in *Saccharomyces cerevisiae* for increasing NADPH production capacity aerobically in different cellular compartments. Metab Eng. 2004;6(4):352–63. 10.1016/j.ymben.2004.06.002 15491864

[37] Gietz D, St Jean A, Woods RA, Schiestl RH (1992). Improved method for high efficiency transformation of intact yeast cells. Nucleic Acids Res.

[38] Hasunuma T, Sanda T, Yamada R, Yoshimura K, Ishii J, Kondo A (2011). Metabolic pathway engineering based on metabolomics confers acetic and formic acid tolerance to a recombinant xylose-fermenting strain of *Saccharomyces cerevisiae*. Microb Cell Fact.

[39] Ishii J, Yoshimura K, Hasunuma T, Kondo A (2013). Reduction of furan derivatives by overexpressing NADH-dependent Adh1 improves ethanol fermentation using xylose as sole carbon source with *Saccharomyces cerevisiae* harboring XR-XDH pathway. Appl Microbiol Biotechnol.

